# Outcomes of high-compliance bracing in patients with severe adolescent idiopathic scoliosis declining operative intervention

**DOI:** 10.3389/fped.2026.1851816

**Published:** 2026-07-24

**Authors:** Dingqi You, Xingyu Duan, Wei Guo, Zhenxing Hou, Jianchao Wang, Weichao Sheng, Wensheng Liao

**Affiliations:** 1 Department of Spinal Cord Surgery, People’s Hospital of Zhengzhou University, Henan Provincial People’s Hospital, Zhengzhou, China; 2 Henan Xinzhi Medical Technology Company Limited, Zhengzhou, China

**Keywords:** adolescent idiopathic scoliosis, bracing, conservative management, surgery refusal, treatment compliance

## Abstract

**Background:**

Surgery is typically recommended for patients with severe adolescent idiopathic scoliosis (AIS); however, some patients explicitly refuse due to concerns about surgical risks and other factors. Conventional wisdom suggests that bracing has limited effectiveness for severe cases, yet evidence for this specific population remains insufficient.

**Methods:**

This single-center retrospective cohort study included patients with severe AIS (Cobb angle >40°) who met surgical indications but refused surgery during the study period (January 2021 to April 2025). All patients received custom-fitted Chêneau braces; a dual-verification compliance was monitored using a dual-verification approach (structured daily diaries cross-checked against physician-assessed skin indentation grading). Multivariate linear regression was used to analyze predictors of treatment outcomes.

**Results:**

A total of 82 patients were enrolled, with a mean follow-up of 14.4 ± 2.1 months. The overall correction rate was 18.0% ± 14.0%, with 56.1% of patients achieving a Cobb angle below 40°, and 39.0% showing improvement greater than 10°. Multivariate analysis revealed that daily brace-wearing time was an independent predictor of correction rate (each additional hour increased the correction rate by 1.88%, *P* = 0.003). In the high-compliance group (>16 h/day), 71.7% of patients achieved a final Cobb angle below 40°. The four core domains of the SRS-22r quality-of-life questionnaire (function/activity, pain, self-image, and mental health) demonstrated significant improvement, and treatment satisfaction was high at final follow-up. Patient motivation showed a strong positive correlation with wearing time (*r* = 0.654). Complications were infrequent**.**

**Conclusions:**

For patients with severe AIS who refused surgery, conservative bracing achieved satisfactory short-to-mid-term outcomes with a favorable safety profile within a shared decision-making framework. Actual daily wearing time demonstrated the strongest independent association with radiographic outcomes among measured variables, while patients' intrinsic motivation showed a strong correlation with compliance, suggesting a potential indirect pathway to improved outcomes. It should be emphasized that this study did not establish bracing as equivalent or superior to surgical correction in severe AIS.

## Introduction

Adolescent Idiopathic Scoliosis (AIS) is the most common three-dimensional spinal deformity, with a global incidence of approximately 0.5%–5.2% (1, 2). When the major curve Cobb angle progresses beyond 40°, surgical intervention is typically indicated to prevent further deformity deterioration (3–5). However, clinicians frequently encounter patients or their guardians who explicitly refuse surgery and strongly prefer conservative treatment due to concerns regarding surgical risks, anesthesia-related complications, postoperative rehabilitation duration, or financial burden (6–9). For this specific population of patients with severe AIS who meet surgical indications yet explicitly refuse operative intervention, the clinical benefit of bracing treatment remains uncertain and warrants rigorous evaluation.

**Figure 1:**
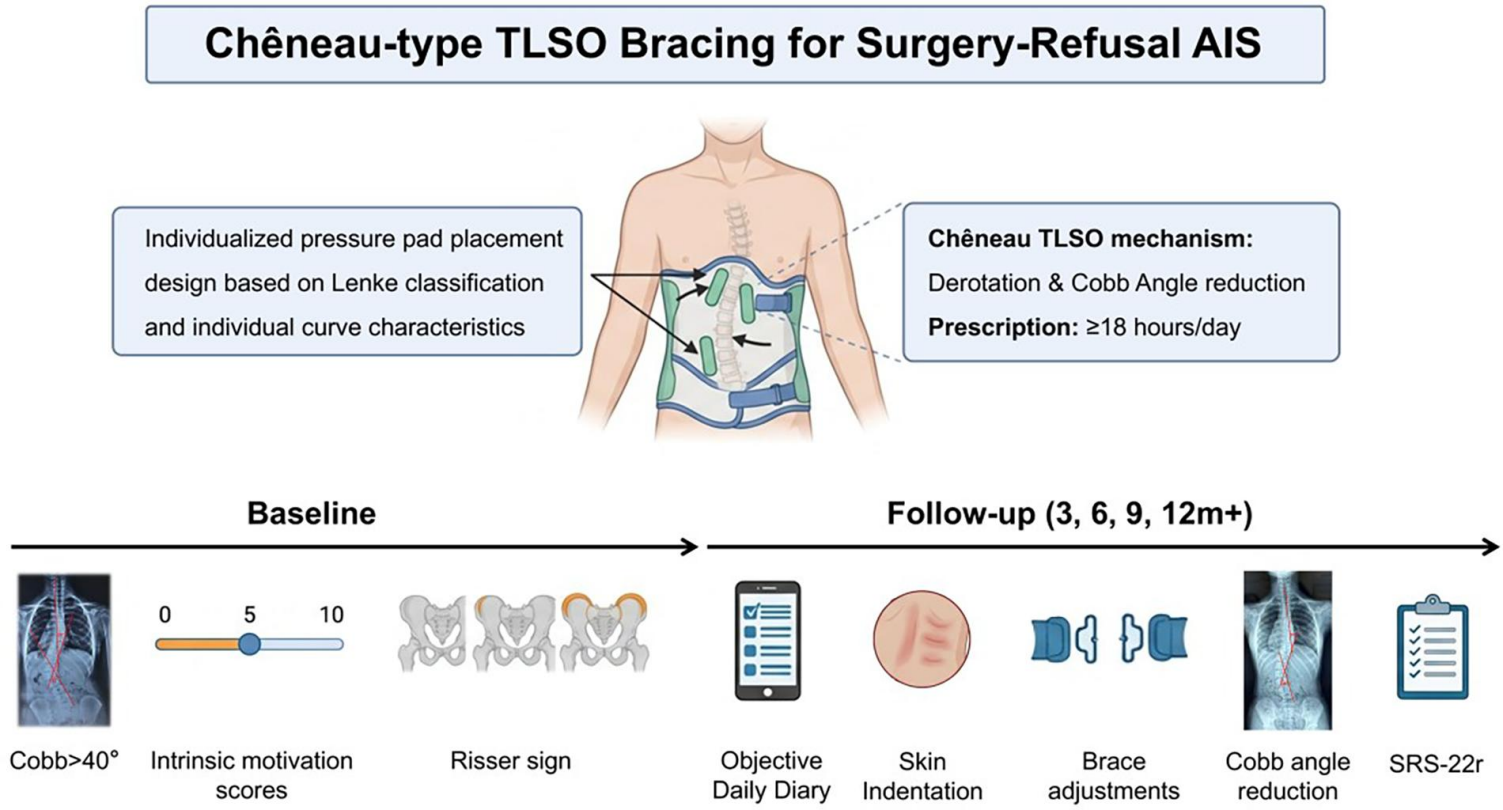
Schematic of the chêneau bracing protocol and follow-up for patients with severe AIS declining surgery.

Conventional clinical wisdom holds that once scoliosis progresses to the severe stage (Cobb angle >40°), brace treatment is of limited effectiveness and may delay the optimal timing for surgery (10, 11). Nevertheless, recent studies have suggested a significant “dose-response” relationship between brace efficacy and daily wearing time (12, 13). For this specific subgroup, the effectiveness, safety, and key influencing factors of bracing therapy have not been fully characterized. Therefore, this study aimed to evaluate the radiographic outcomes and associated factors of conservative bracing within a shared decision-making framework for patients with severe AIS who meet surgical indications yet explicitly refuse operative intervention. Additionally, we explored the relationship between daily wearing time, patient motivation, and radiographic improvement. A schematic overview of the study design is shown in Figure 1.

## Materials and methods

### Study population

This study was conducted from January 2021 to April 2025. Patients with severe AIS (major curve Cobb angle >40° on standing full-spine radiographs combined with Risser sign 0–4, indicating remaining growth potential and progression risk) who met surgical indications but explicitly declined operative intervention after detailed counseling were eligible for inclusion. Surgical indications were confirmed through independent assessments by two senior orthopedic surgeons. Curve progression velocity, symptom severity, or specific Lenke types were not used as independent surgical indication criteria.

#### Inclusion criteria

(1) Age 10–18 years; (2) Standing full-spine radiographs demonstrating a major curve Cobb angle greater than 40°; (3) Risser sign 0–4; (4) Explicit refusal of surgery after detailed preoperative counseling (including surgical risks, prognosis, and limitations of conservative treatment); (5) Voluntary acceptance of conservative brace treatment with a commitment to follow-up.

#### Exclusion criteria

History of previous spinal surgery, congenital vertebral anomalies, Marfan syndrome or other connective tissue disorders, follow-up duration less than 12 months, or incomplete imaging data.

This single-center retrospective cohort study was approved by the Ethics Committee of Henan Provincial People's Hospital; all data were extracted from the institutional electronic medical record system and routine scoliosis follow-up registry. We clarify that all documentation was generated during routine clinical care, not for research purposes. Written informed refusal of surgery and written informed consent for brace therapy were standard clinical requirements for patients who declined operative intervention, with all records archived as routine medical documentation. SRS-22r assessments and compliance monitoring were also administered at every follow-up visit per the standardized brace-management protocol, ensuring complete data capture without additional research-specific procedures.

### Baseline assessment protocol

All patients underwent standardized evaluation before brace customization and at the final follow-up, encompassing four domains: imaging examination, physical examination, quality-of-life assessment, and treatment motivation assessment.

Full-length standing anteroposterior and lateral radiographs of the spine were obtained. Two senior orthopedic surgeons independently measured the major curve Cobb angle blinded to one another's measurements (mean values were used; intraclass correlation coefficient [ICC] >0.90), and Lenke classification was applied. Flexibility was assessed using supine side-bending radiographs, calculated as: Flexibility Index = [(Standing Cobb angle−Side-bending Cobb angle)/Standing Cobb angle] × 100%. Skeletal maturity was evaluated using the Risser sign (grades 0–4). For female patients, menstrual status was recorded (pre-menarche, within 1 year post-menarche, or >1 year post-menarche). The SRS-22r questionnaire (Scoliosis Research Society-22r) was administered at baseline and final follow-up (14–16). This instrument comprises four domains (function/activity, pain, self-image, and mental health, with 5 items each) and a separate treatment satisfaction item, utilizing a 5-point Likert scale (1–5). Higher total and domain scores indicate better quality of life.

Following the standard clinical consent process for brace treatment and explicit refusal of surgery documented in the medical record, independent researchers conducted a baseline motivation intensity assessment using a 10-point Visual Analog Scale (VAS). The assessment was performed in a standardized quiet consultation room. Patients were presented with a horizontal line with the left end labeled “0 = fully accept surgery, no willingness for conservative treatment” and the right end labeled “10 = extremely strong refusal of surgery, strong preference for conservative treatment.” Patients marked their willingness on the line, and researchers measured the distance precisely (recorded to one decimal place). This assessment was completed before brace fitting, and the researchers were blinded to subsequent compliance monitoring staff to avoid observer bias.

### Brace intervention protocol

All patients received custom-fitted Chêneau-type thoracolumbosacral orthoses (TLSO) by the same senior orthotist (17, 18). Based on Lenke classification and individual curve patterns, pressure pads were individually designed on the convex side of the curve to achieve coronal plane correction while maintaining sagittal physiological curvature. The prescription required daily wear for ≥18 h. Standardized skin care protocols and guidance for core muscle training during brace-free periods were provided. Actual compliance was recorded through the monitoring system described in “Compliance monitoring system”.

### Compliance monitoring system

A dual-verification compliance monitoring framework was established to ensure objective measurement of wearing time. It should be emphasized that this framework does not utilize electronic temperature or pressure sensors. Primary monitoring: Patients completed structured online brace-wearing diaries daily, recording actual hours worn. Secondary monitoring: During monthly outpatient reviews, attending physicians independently verified cumulative wearing duration by examining the degree of skin indentation in the thoracolumbar region (graded 0–3: grade 0 = no indentation; grade 1 = resolves within 30 min; grade 2 = resolves between 30 and 60 min; grade 3 = persists beyond 60 min), and cross-checked the findings against diary records. This dual-verification approach reduces—but does not eliminate—subjectivity inherent in self-reported data.

### Follow-up protocol

Patients attended monthly outpatient visits after brace fitting. Full-length standing anteroposterior and lateral radiographs were obtained at 3, 6, 9, and 12 months after a washout period of approximately 12 h (patients were instructed to remove the brace at bedtime the night before their scheduled morning appointment). The major curve Cobb angle was measured independently by two senior orthopedic surgeons blinded to each other's measurements; mean values were used for analysis, with inter-observer consistency verified by ICC >0.90. Each monthly visit included clinical assessment, physical examination, brace fit adjustment, and compliance evaluation.

### Outcome measures

The primary outcome was the absolute change in major curve Cobb angle from baseline to final follow-up (mean 14.4 ± 2.1 months). Secondary outcomes included: (1) Correction rate (Cobb angle change/baseline Cobb angle×100%); (2) Proportion of patients achieving curve improvement >10° or final Cobb angle <40°; (3) SRS-22r quality-of-life scores across five dimensions: function/activity, pain, self-image, mental health, and treatment satisfaction; (4) Compliance-stratified subgroup analysis results; (5) Complication incidence.

### Statistical methods

Continuous variables are expressed as mean ± standard deviation; categorical variables are expressed as frequency (percentage). Multivariate linear regression was used to adjust for confounding factors including baseline Cobb angle, Risser sign, Lenke classification (with Type 1 as reference and dummy variables set), and initial flexibility index, to evaluate the independent associations between variables and radiographic correction rate. Age, sex, and menstrual status were not included in the final model: age showed limited variance (13.9 ± 1.9 years) and was highly correlated with Risser sign; the sample was predominantly female (76.8%), restricting sex-based comparisons; and menstrual status was collinear with Risser sign (both reflecting skeletal maturity). In-brace correction rate, apical vertebral rotation, PSSE exposure, and family support were not consistently documented in retrospective medical records and thus could not be incorporated.

Patients were stratified into three compliance subgroups based on actual daily wearing time: high compliance (>16 h/day), moderate compliance (10–16 h/day), and low compliance (<10 h/day), with between-group differences analyzed using analysis of variance (ANOVA). Pearson correlation analysis assessed the relationship between patients’ intrinsic motivation to avoid surgery (baseline 10-point VAS) and actual wearing time, with stratified analysis employed to explore their joint effects on treatment outcomes. Two-sided *P* < 0.05 was considered statistically significant. All statistical analyses were performed using SPSS 26.0 software (IBM Corporation, Armonk, NY, USA).

## Results

### Baseline characteristics of study participants

**Figure 2:**
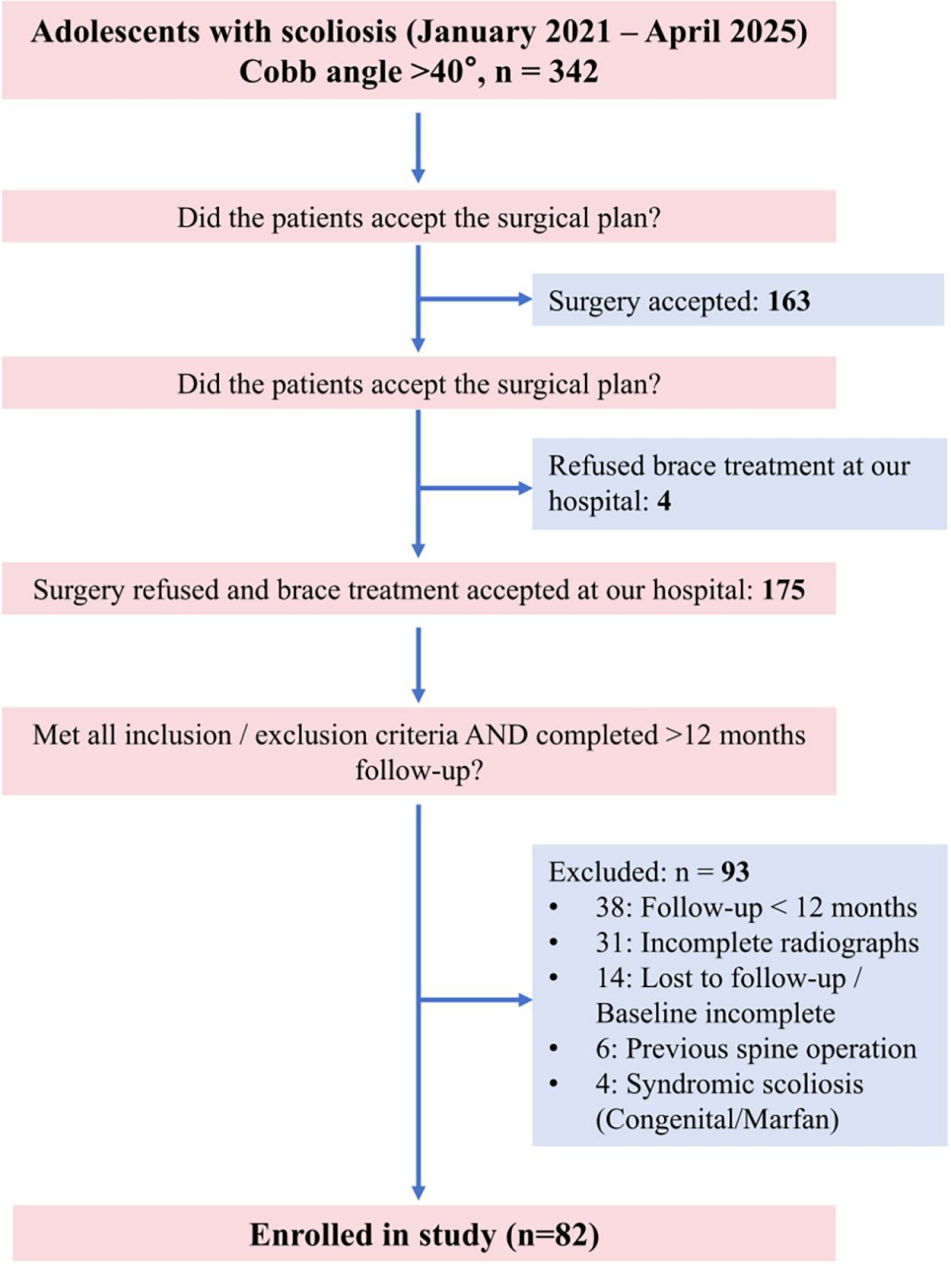
Flowchart of patient screening, exclusion criteria, and final enrollment for bracing in patients with severe AIS who declined surgery.

During the study period, 342 patients with AIS who met surgical indications were screened. Of the 260 patients not included in the final analysis, 163 opted for surgical treatment after detailed preoperative counseling, and 4 declined surgery but chose to receive bracing at other institutions. The remaining 93 patients agreed to conservative management at our center but were excluded based on prespecified criteria: 38 had a follow-up duration <12 months, 31 had incomplete imaging data, 14 were lost to follow-up or had incomplete baseline assessment, 6 had a history of previous spinal surgery, and 4 had congenital vertebral anomalies or connective tissue disorders (e.g., Marfan syndrome). Ultimately, 82 patients who refused surgery and accepted conservative treatment were enrolled (screening flowchart shown in Figure 2). The baseline characteristics of the enrolled patients are summarized in Table 1 and described below.

**Table 1:** Baseline demographic, radiographic, skeletal maturity, and treatment characteristics of 82 patients with severe AIS declining surgery.

Characteristic	Value (Mean ± SD or *n* (%))	Range/Notes
Age (years)	13.9 ± 1.9	10–18
Gender, *n* (%)		
Female	63 (76.8)	
Male	19 (23.2)	
Disease Characteristics		
Baseline major curve Cobb angle (°)	47.7 ± 3.9	40.4–55.0
Risser sign, *n* (%)		
Grade 0–2 (high growth potential)	59 (72.0)	
Grade 3–4 (skeletal maturity)	23 (28.0)	
Lenke classification, *n* (%)		
Type 1 (Main thoracic)	39 (47.6)	
Type 3 (Double major)	28 (34.1)	
Type 5 (Thoracolumbar/Lumbar)	15 (18.3)	
Flexibility index (%)	29.7 ± 11.5	11.0–52.0
Menstrual Status (*n* = 63)		
Premenarche	24 (38.1)	
≤1 year post-menarche	15 (23.8)	
>1 year post-menarche	24 (38.1)	
Treatment-Related Characteristics		
Surgery avoidance motivation score (0–10)	7.4 ± 1.4	4–9
Actual daily wearing time (hours)	14.8 ± 3.1	8.2–18.4

Data are presented as mean ± standard deviation or frequency (percentage). Total sample size *n* = 82.

**Figure 3:**
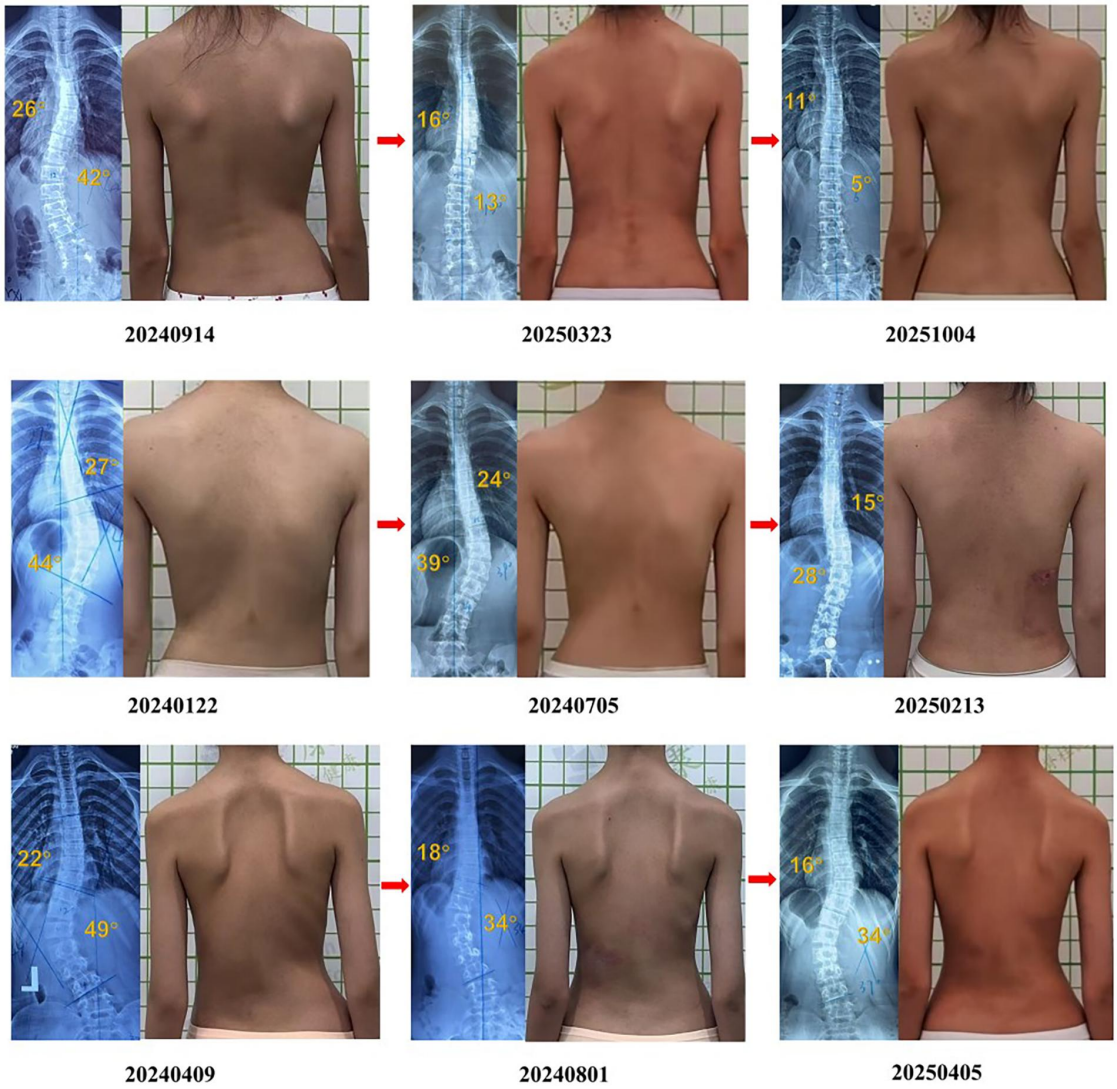
Representative cases demonstrating radiographic cobb angle and clinical surface deformity changes at baseline and after chêneau bracing in patients with severe AIS who declined surgery.

The mean age was 13.9 ± 1.9 years, with 63 females and 19 males. The mean baseline major curve Cobb angle was 47.7° ± 3.9°. Fifty-nine patients had Risser sign 0–2, and 23 had Risser sign 3–4. Lenke classification distribution was as follows: Type 1 in 39 patients, Type 3 in 28, and Type 5 in 15. The mean flexibility index was 29.7% ± 11.5%. Surface appearance and radiographic changes of a representative case are shown in Figure 3.

To further explore the clinical relevance of the initial flexibility index, patients were stratified into high-flexibility (>30%) and low-flexibility (≤30%) subgroups based on the cohort median. The high-flexibility group (*n* = 41) demonstrated a mean correction rate of 21.3 ± 15.2%, compared with 14.9 ± 12.1% in the low-flexibility group (*n* = 41). Although this difference did not reach statistical significance in univariate analysis (*t* = 1.98, *P* = 0.051), the effect size suggested a clinically meaningful trend favoring greater initial flexibility. Notably, Lenke Type 5 patients showed a lower mean flexibility index (24.8 ± 9.3%) than Lenke Type 1 (31.5 ± 11.8%) and Type 3 (29.1 ± 10.4%), which may partially account for the inferior correction outcomes observed in this subgroup. In the multivariate model, initial flexibility index did not emerge as an independent predictor (Table 4), likely due to collinearity with Risser sign and Lenke classification.

### Radiographic outcomes of brace treatment

**Figure 4:**
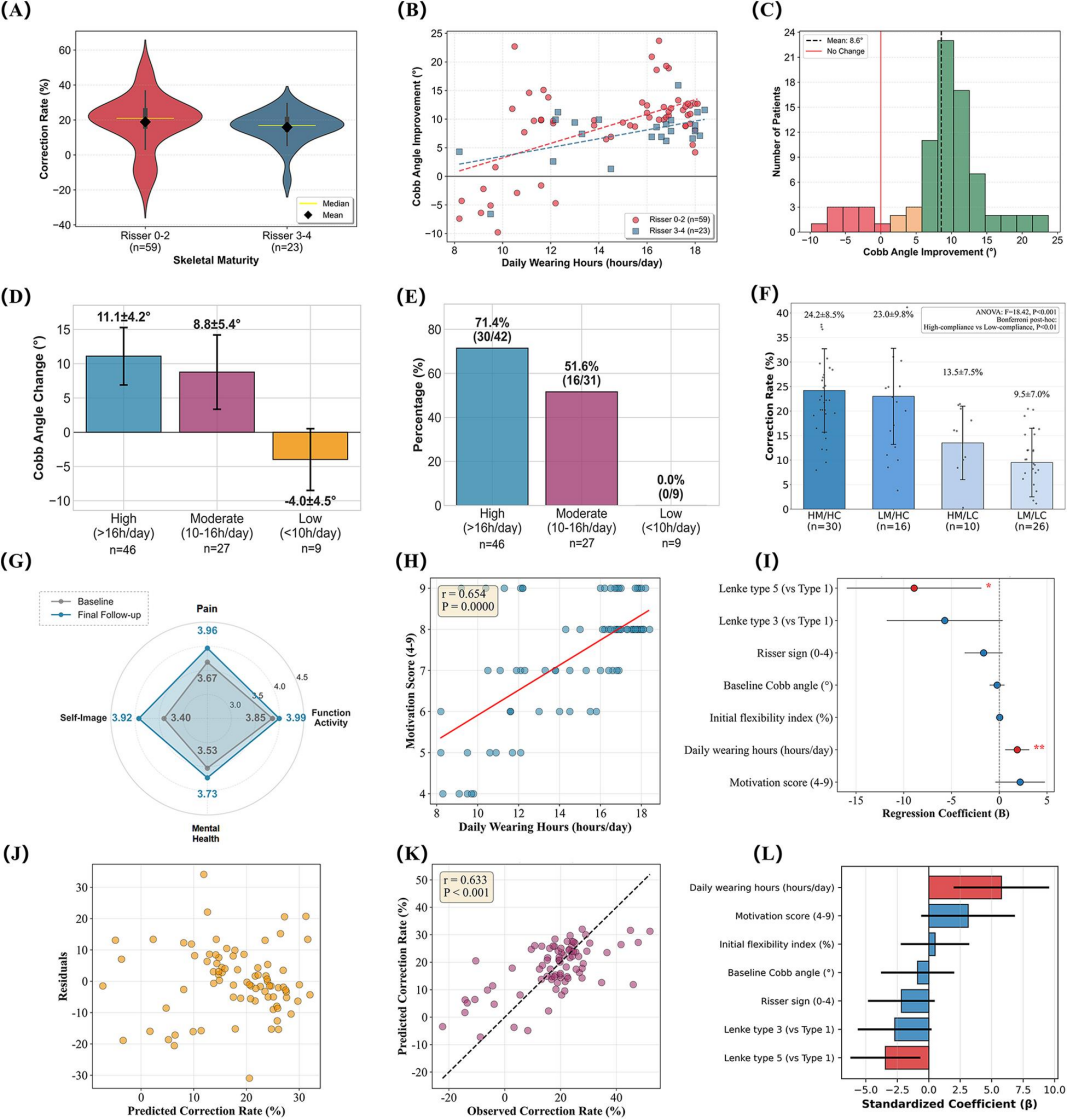
Comprehensive analysis of brace treatment outcomes, compliance, and quality of life in patients with severe AIS who declined surgery. **(A)** Violin plot of correction rate distribution by skeletal maturity subgroups (Risser 0–2 vs. 3–4); **(B)** Scatter plot of the correlation between daily wearing time and Cobb angle improvement (stratified by skeletal maturity); **(C)** Histogram of Cobb angle improvement values; **(D)** Bar chart of Cobb angle changes across different compliance groups; **(E)** Proportion of patients achieving Cobb angle <40° in each compliance group; **(F)** Correction rates across four motivation–compliance subgroups; **(G)** Radar chart of SRS-22r quality-of-life domain scores (baseline vs. final follow-up); **(H)** Correlation between daily wearing time and motivation score for avoiding surgery; **(I)** Forest plot of multivariate linear regression coefficients; **(J)** Residual plot; **(K)** Scatter plot of observed vs. predicted values; **(L)** Comparison plot of standardized regression coefficients. Error bars represent 95% confidence intervals or standard deviations.

Radiographic assessment results are presented in Table 2. All follow-up radiographs were obtained after a standardized brace-free interval of approximately 12 h to eliminate passive in-brace correction effects. At final follow-up, the mean major curve Cobb angle decreased by 8.6 ± 6.5° (t = 11.88, *P* < 0.001), with an overall correction rate of 18.0 ± 14.0%. Cobb angle decreased below 40° in 56.1% of patients, meeting non-operative management criteria; improvement exceeding 10° was observed in 39.0% of patients. Stratified analysis by Risser sign (Figure 4A) revealed that patients with greater growth potential (Risser 0–2) achieved numerically higher correction rates than skeletally mature patients (Risser 3–4), although the between-group difference did not reach statistical significance (*t* = 0.88, *P* = 0.382). Patients with Lenke Type 1 curves demonstrated higher correction rates (19.9 ± 17.1%) than those with Type 3 (18.1 ± 6.9%) and Type 5 (13.2 ± 14.5%), suggesting potential differences in treatment outcomes among curve types—subsequent multivariate analysis further verified their independent effects. Notably, curve progression occurred in 12.2% (10/82) of patients (Figure 4C); however, no patient progressed to require urgent surgical intervention, and the majority of progressive cases were of modest magnitude.

**Table 2:** Radiographic outcomes of brace treatment at final follow-up by maturity, lenke, and compliance in patients with severe AIS.

Group	*n*	Cobb Change (°)	Correction Rate (%)	Cobb <40° (%)	Improvement >10° (%)	Progression (%)	Statistics
Overall	82	8.6 ± 6.5	18.0 ± 14.0	56.1 (46/82)	39.0 (32/82)	12.2 (10/82)	*t* = 11.88, *P* < 0.001
Risser 0–2	59	9.0 ± 7.2	18.9 ± 15.5	59.3 (35/59)	47.5 (28/59)	15.3 (9/59)	
Risser 3–4	23	7.6 ± 4.3	15.9 ± 9.1	47.8 (11/23)	17.4 (4/23)	4.3 (1/23)	*t* = 0.88, *P* = 0.382
Lenke 1 (MT)	39	9.3 ± 8.0	19.9 ± 17.1	61.5 (24/39)	46.2 (18/39)	15.4 (6/39)	
Lenke 3 (DM)	28	8.8 ± 3.3	18.1 ± 6.9	46.4 (13/28)	39.3 (11/28)	3.6 (1/28)	1 vs. 3: *t* = 0.52, *P* = 0.607
Lenke 5 (TL/L)	15	6.1 ± 6.6	13.2 ± 14.5	60.0 (9/15)	20.0 (3/15)	20.0 (3/15)	1 vs. 5: *t* = 1.32, *P* = 0.191
High Compliance (>16 h)	46	11.2 ± 4.0	23.8 ± 9.0	71.7 (33/46)	58.7 (27/46)	0.0 (0/46)	
Moderate Compliance (10–16 h)	27	8.3 ± 5.6	17.1 ± 11.8	48.1 (13/27)	18.5 (5/27)	11.1 (3/27)	
Low Compliance (<10 h)	9	−4.0 ± 4.5	−8.5 ± 9.5	0.0 (0/9)	0.0 (0/9)	77.8 (7/9)	*F* = 37.79, *P* < 0.001

### Compliance-stratified treatment outcomes and association with patient motivation

Compliance monitoring data revealed a mean actual daily wearing time of 14.8 ± 3.1 h. As shown in Figure 4D, the high-compliance group (>16 h/day, *n* = 46) achieved a mean Cobb angle improvement of 11.1 ± 4.0°, superior to the moderate-compliance group (10–16 h/day, *n* = 27, 8.3 ± 5.6°) and the low-compliance group (<10 h/day, *n* = 9, −4.0 ± 4.5°). In the high-compliance group, 71.7% of patients achieved a final Cobb angle <40°, compared with only 48.1% in the moderate-compliance group and no patients in the low-compliance group (Figure 4E). The mean baseline motivation score was 7.4 ± 1.4 points (range: 4–9). Figure 4B demonstrates a positive correlation between daily wearing time and Cobb angle improvement, with an overall correlation coefficient *r* = 0.52, *P* < 0.001; stratified analysis showed a steeper slope in Risser 0–2 patients, suggesting greater sensitivity to wearing time among those with higher growth potential. To avoid methodologically inappropriate nested comparisons, patients were stratified into four non-overlapping subgroups by motivation score (≥8 vs. < 8) and daily wearing time (>16 vs. ≤16 h/day): high motivation/high compliance (HM/HC; *n* = 30, correction rate 24.2 ± 8.5%), low motivation/high compliance (LM/HC; *n* = 16, 23.0 ± 9.8%), high motivation/low compliance (HM/LC; *n* = 10, 13.5 ± 7.5%), and low motivation/low compliance (LM/LC; *n* = 26, 9.5 ± 7.0%). One-way ANOVA with Bonferroni *post-hoc* correction revealed significant between-group differences (*F* = 18.42, *P* < 0.001). High-compliance subgroups collectively demonstrated significantly higher correction rates than low-compliance subgroups (Bonferroni post-hoc, *P* < 0.01), whereas no significant difference was detected between the HM/HC and LM/HC groups (*P* = 0.412), suggesting that daily wearing time, rather than intrinsic motivation *per se*, was more strongly associated with radiographic improvement (Figure 4F).

### Quality of life improvements

Quality-of-life assessments are presented in Table 3. The total SRS-22r score improved from 3.61 ± 0.28 at baseline to 3.96 ± 0.31 at final follow-up (t = −17.13, *P* < 0.001). The radar chart (Figure 4G) demonstrates that the self-image domain showed the most significant improvement (3.40 ± 0.72 vs. 3.92 ± 0.74, *t* = −16.08, *P* < 0.001), with notable improvements also observed in the pain domain (3.67 ± 0.54 vs. 3.96 ± 0.61, *t* = −7.95, *P* < 0.001). Improvements were also observed in the function/activity domain (3.85 ± 0.63 vs. 3.99 ± 0.65, *P* < 0.001) and mental health domain (3.53 ± 0.71 vs. 3.73 ± 0.78, *P* < 0.001). The treatment satisfaction domain had a mean score of 4.12 ± 0.55 points, indicating good patient acceptance of this conservative strategy.

**Table 3:** SRS-22r quality of life scores at baseline and final follow-up in patients with severe AIS.

Dimension	Baseline_Mean_SD	Final_Mean_SD	Mean_Difference	t_value	*P*_value
Function/Activity	3.85 ± 0.63	3.99 ± 0.65	0.14	−5.03	<0.001
Pain	3.67 ± 0.54	3.96 ± 0.61	0.29	−7.95	<0.001
Self-Image	3.40 ± 0.72	3.92 ± 0.74	0.52	−16.08	<0.001
Mental Health	3.53 ± 0.71	3.73 ± 0.78	0.20	−5.08	<0.001
Total Score	3.61 ± 0.28	3.96 ± 0.31	0.35	−17.13	<0.001
Treatment Satisfaction	–	4.12 ± 0.55	–	–	–

Negative t-values indicate score increases (improvement) from baseline to final follow-up based on the calculation direction (baseline minus final).

**Table 4:** Multivariate linear regression analysis of independent predictors of radiographic correction rate in patients with severe AIS.

Predictor	B (Coefficient)	SE	95% CI Lower	95% CI Upper	Standardized *β*	*t*	*P* value formatted	Sig.
Intercept	−9.762	19.063	−47.745	28.221	Reference	−0.512	0.610	NS
Baseline Cobb angle (°)	−0.231	0.368	−0.964	0.503	−0.064	−0.626	0.533	NS
Risser sign (0–4)	−1.641	0.973	−3.580	0.298	−0.120	−1.686	0.096	NS
Initial flexibility index (%)	0.043	0.116	−0.187	0.274	0.035	0.375	0.709	NS
Daily wearing hours (hours/day)	1.877	0.607	0.668	3.085	0.416	3.093	0.003	**
Motivation score (4–9)	2.179	1.275	−0.362	4.720	0.225	1.709	0.092	NS
Lenke type 3 (vs. Type 1)	−5.713	3.017	−11.725	0.298	−0.195	−1.894	0.062	NS
Lenke type 5 (vs. Type 1)	−8.917	3.512	−15.914	−1.919	−0.248	−2.539	0.013	*

Data are presented as B (unstandardized coefficient), SE, 95% confidence interval, standardized β, t, and *P* values. Standardized β for continuous variables was calculated as B × (SD_predictor/SD_outcome); for categorical variables (Lenke type 3 and 5), standardized β was derived from SPSS output using dummy variable coding with Type 1 as the reference.

* *P* < 0.05.

** *P* < 0.01.

### Independent predictors of radiographic outcomes

To identify independent predictors of brace treatment outcomes, this study employed multivariate linear regression analysis (Table 4). The overall model was statistically significant (*F* = 7.077, *P* < 0.001), explaining 34.4% of the variance in radiographic correction rate (adjusted R^2^ = 0.344). Figure 4K shows a positive correlation between observed and predicted correction rates (*r* = 0.633, *P* < 0.001), with data points distributed along the diagonal, indicating good model predictive accuracy. Figure 4L presents standardized regression coefficients for each predictor, revealing that daily wearing time had the largest effect size, followed by Lenke Type 5. After controlling for baseline Cobb angle, Risser sign, initial flexibility index, and Lenke classification, daily wearing time emerged as an independent predictor of correction rate (Figure 4I)—each additional hour of daily wearing time was associated with a mean increase of 1.88% in radiographic correction rate. Furthermore, patients with Lenke Type 5 curves demonstrated 8.92% lower correction rates compared with Type 1, suggesting differential treatment responses across curve types. Notably, although the unadjusted between-group comparison in Table 2 did not reach significance for Lenke Type 5 vs. Type 1 (*t* = 1.32, *P* = 0.191), this discrepancy reflected confounding by other baseline prognostic factors (including Cobb angle and skeletal maturity); once these were adjusted in the multivariate model, the independent negative effect of Lenke Type 5 became statistically evident. However, motivation scores (*P* = 0.092), Risser sign (*P* = 0.096), and Lenke Type 3 (*P* = 0.062) did not reach conventional significance thresholds.

Furthermore, motivation scores showed a strong positive correlation with daily wearing time (*r* = 0.654, *P* < 0.001) (Figure 4H). This correlation was significant only in univariate analysis, with the effect of motivation mediated by wearing time in the multivariate model, suggesting that wearing time may represent a critical mediating pathway through which motivation influences treatment outcomes. Model diagnostics indicated approximately normal distribution of residuals (Figure 4J), suggesting good model fit.

### Safety and complications

Regarding safety, mild skin indentations occurred in 20.7% (17/82) of patients (Grade 1 in 12 cases, Grade 2 in 5 cases), all representing reversible changes. Localized Grade 1 pressure ulcers occurred in 2.4% (2/82) of patients (both located at the sternal prominence), which resolved within 1 week following brace adjustment and skin care. No severe complications such as respiratory dysfunction, neurological injury, or secondary infection of pressure ulcers were observed. All 82 patients completed follow-up through the final assessment without attrition.

## Discussion

This single-center retrospective cohort study systematically evaluated the radiographic outcomes, safety, and associated factors of conservative bracing in patients with severe AIS who met surgical indications but explicitly refused surgery. A total of 82 patients were enrolled, and after a mean follow-up of 14.4 months, the results demonstrated an overall correction rate of 18.0%, with 56.1% of patients achieving a Cobb angle below 40° and 39.0% showing improvement exceeding 10°. It is critical to emphasize that the evidence provided herein is limited to the mean follow-up duration of 14.4 months and cannot be extrapolated to long-term curve stability, post-maturity progression, or ultimate surgical conversion rates. Furthermore, this study did not establish bracing as equivalent or superior to surgical correction in severe AIS; rather, it provided evidence-based guidance for a specific subgroup of patients who definitively declined operative intervention, supporting shared decision-making within a conservative management framework. Given the absence of a surgical or observation control group, these observed radiographic changes should be interpreted as associations rather than confirmed causal intervention effects, and we cannot exclude contributions from the natural history of the disease or regression to the mean. The four core domains of the SRS-22r questionnaire (function/activity, pain, self-image, and mental health) demonstrated significant improvement, and treatment satisfaction was high at final follow-up. Multivariate linear regression analysis revealed that, after controlling for baseline Cobb angle, Risser sign, flexibility index, and Lenke classification, actual daily wearing time emerged as the strongest independent modifiable predictor of radiographic correction rate (each additional hour of wearing time increased the correction rate by 1.88 percentage points, *P* = 0.003), whereas Lenke Type 5 (thoracolumbar/lumbar curve) showed a significant independent negative effect compared with Lenke Type 1 (main thoracic curve) (*P* = 0.013). Notably, although patients' intrinsic motivation to avoid surgery showed a strong positive correlation with wearing time (*r* = 0.654, *P* < 0.001), motivation scores did not demonstrate independent predictive value in the multivariate model (*P* = 0.092), with its effect potentially mediated by wearing time, although formal mediation analysis was not performed. This suggested a plausible explanatory model in which motivation enhanced compliance (i.e., extending wearing time), which in turn might improve outcomes. Causal inference cannot be established from these correlational data. We acknowledge that the observed correlation between motivation and wearing time (*r* = 0.654), and between wearing time and radiographic improvement, did not constitute proof of a mediating pathway; future prospective studies employing formal mediation analysis (e.g., Baron–Kenny approach or structural equation modeling) are required to validate this hypothetical mechanism.

The threshold of >16 h/day for defining high compliance warranted methodological clarification. This cutoff was selected as a pragmatic, data-driven stratification based on the observed distribution of actual wearing time in this cohort (mean 14.8 ± 3.1 h, range 8.2–18.4 h). Although SOSORT guidelines (19) recommend a target of ≥18 h/day, real-world adherence among patients with severe AIS rarely achieved this ideal due to academic and psychosocial demands. Thus, >16 h/day represented the most clinically attainable high-adherence level closest to the prescribed target that was sustainable over the long term, while maintaining a clear statistical gradient from the moderate-compliance group. We acknowledge that this threshold was not derived from an externally validated literature standard; rather, it reflected a pragmatic choice driven by our data distribution, offering a realistic benchmark for clinical practice.

The value of bracing for severe AIS (Cobb angle >40°) has long been controversial in the published literature. Traditional viewpoints suggest that when scoliosis progresses to the range of surgical indications, brace treatment is often ineffective and may delay surgical timing, leading to deformity deterioration (20–22). However, recent evidence suggests that for patients with severe scoliosis (particularly those who are skeletally immature), high-quality brace wear may still yield clinical benefits (23–25). Babaee et al. (7) conducted a meta-analysis and found that brace treatment had significant effects on controlling progression of severe curves, with a mean improvement rate of 38.8%, stabilization rate of 25.5%, and progression rate of 35.4%. Factors influencing treatment efficacy included Risser staging (lower skeletal maturity associated with poorer outcomes), initial curve severity, in-brace correction rate, and apical vertebral rotation. Rahman et al. (26) reported that patients with 85% compliance achieved significantly better outcomes than those with 62% compliance, highlighting the importance of compliance. This study utilized the widely used Chêneau-type TLSO, while other investigators have studied alternative brace designs (27). Zhang et al. (28) employed modified Gensingen bracing combined with exercise training in patients with AIS (Cobb angles of 40°–60°) who refused surgery. Their results showed that the mean major curve decreased from 47.40° at baseline to 38.56° at final follow-up (*P* < 0.001), with 65% of patients demonstrating curve improvement, 30% stabilization, and 5% progression. Their study similarly provided evidence for non-surgical treatment options in patients with severe scoliosis. The present study enrolled only Lenke Types 1, 3, and 5 patients, excluding Lenke Type 2 (double thoracic), Type 4 (single lumbar), and Type 6 (thoracolumbar major) curves. The rationale was as follows: Lenke Types 2 and 6 represent double thoracic or thoracolumbar major curves, which are difficult to control simultaneously by bracing; Lenke Type 4 represents single lumbar curves, for which brace treatment is often ineffective due to high lumbar mobility and limited transmission of corrective forces, and such patients are generally more suitable for surgical treatment (29–31).

After multivariate adjustment, Lenke Type 5 curves demonstrated significantly lower correction rates than Type 1. While anatomical biomechanics (high lumbar mobility and limited counterforce transmission at the lumbosacral junction) partially explain this finding, we further propose that the Duval-Beaupère model may provide deeper mechanistic insight (32). This model conceptualizes scoliotic deformity as comprising three structural components: postural, elastic, and rigid. The initial flexibility index, measured in this study by the supine side-bending radiograph, serves as a clinical proxy for the elastic component—the proportion of deformity amenable to reversible correction. In this cohort, Lenke Type 5 patients exhibited a lower mean flexibility index (24.8 ± 9.3%) compared with Type 1 (31.5 ± 11.8%) and Type 3 (29.1 ± 10.4%). Although flexibility index did not emerge as an independent predictor in the multivariate model (likely constrained by sample size and collinearity with Risser sign and Lenke classification), its potential mechanistic role should not be dismissed. Patients with a predominantly rigid component (low flexibility) may have limited capacity for sustained structural correction under brace-mediated forces, even with high daily wearing time. This interpretation aligns with the clinical observation that Lenke Type 5 curves, often perceived as more controllable in certain directional patterns, may still respond poorly if the underlying elastic component is insufficient. Future prospective studies should stratify Lenke Type 5 patients by both curve direction and flexibility index to validate this hypothesis and inform tailored brace designs or combined physiotherapeutic scoliosis-specific exercise protocols.

The strengths of this study were threefold. First, the focus on a clinical decision-making challenge—the specific population of patients meeting surgical indications but subjectively refusing surgery, with their families; Second, the establishment of a dual-verification compliance monitoring framework (structured daily diaries cross-checked by physician-verified skin indentation grading) rather than relying solely on subjective questionnaires, which improved the reliability of self-reported wearing time data; Third, in the high-compliance group (>16 h/day) in this study, no patient experienced severe progression (>10°), and the complication rate was extremely low (only 2.4% mild pressure ulcers), confirming the safety of this approach.

Future research should integrate IoT-enabled temperature or pressure sensors for objective, real-time compliance tracking, and prospectively collect data on in-brace correction rates, apical vertebral rotation, and PSSE exposure. Given that 72.0% of patients were skeletally immature at baseline, long-term follow-up (≥5 years) was essential to determine ultimate surgical conversion rates. A multicenter randomized controlled trial comparing conservative bracing vs. immediate surgery in patients who definitively refuse operative intervention would provide the highest level of evidence for this clinical dilemma.

### Limitations of the study

This study has several limitations. First, the population represented a highly selected subgroup of patients with severe AIS who explicitly refused surgery, accepted bracing, and adhered to follow-up. These patients likely differed from the broader population of patients with severe AIS in treatment motivation, family support, and risk perception; our findings should not be generalized to those ambivalent about treatment, those with insufficient family support, or those unable to commit to long-term follow-up. Second, as a single-center retrospective cohort without a surgical or observation control group, this study cannot infer the net effect of bracing through a counterfactual framework; the observed radiographic changes may partly reflect disease natural history or regression to the mean rather than intervention effects alone. Third, the sample size (*n* = 82) was limited, with only 15 Lenke Type 5 cases. With 6 predictor variables fitted to 82 observations, the model operated near the lower limit of acceptable robustness; the adjusted R^2^ of 0.344 indicated that approximately 66% of variance in correction rate remained unexplained. Fourth, the mean follow-up of 14.4 months represented short-to-mid-term outcomes, precluding assessment of long-term curve stability and ultimate surgical conversion rates—this was particularly relevant as 72.0% of patients were skeletally immature (Risser 0–2) at baseline. Fifth, several clinically important variables were unavailable due to retrospective data constraints, including in-brace correction rate, apical vertebral rotation, PSSE exposure, and family support; these omissions not only limited explanatory power but might also have introduced residual confounding. Sixth, motivation was assessed at a single baseline time point without dynamic monitoring. Seventh, the dual-verification framework relied on self-reported diaries and physician-graded skin indentation without electronic sensors for objective tracking. This limitation reflected the retrospective design: IoT sensors were unavailable at our center in 2021 and not covered by insurance, imposing additional costs on patients. Thus, neither systematic overestimation from social desirability bias nor inter-observer variability can be ruled out.

## Conclusion

For patients with severe AIS who met surgical indications but explicitly refused surgery, conservative brace treatment achieved satisfactory short-to-mid-term outcomes (correction rate 18.0%, with 56.1% achieving Cobb angle below 40°) with a favorable safety profile. This study did not establish bracing as equivalent or superior to surgery; rather, it provided evidence-based guidance for shared decision-making when patients definitively declined operative intervention. Actual daily wearing time demonstrated the strongest independent association with radiographic improvement, while intrinsic motivation showed a strong correlation with compliance, suggesting a potential indirect pathway to improved outcomes that warranted formal mediation analysis in future studies. In clinical practice, emphasis should be placed on monitoring and optimizing the actual duration of brace wear.

## Data Availability

The raw data supporting the conclusions of this article will be made available by the authors, without undue reservation.
